# Relative Fat Mass: Refining Adiposity Measurement in the Era Beyond Body Mass Index

**DOI:** 10.1007/s11897-025-00709-w

**Published:** 2025-07-19

**Authors:** Navin Suthahar, Emily S. Lau, Gianluigi Savarese

**Affiliations:** 1 Department of Cardiology, Cardiovascular Research Institute, Thorax Center, Erasmus Medical Center, Rotterdam, The Netherlands; 2 Heart and Vascular Institute, Division of Cardiology, Mass General Brigham, Boston, MA USA; 3 Department of Clinical Science and Education, Södersjukhuset; Karolinska Institutet, Stockholm, Sweden

**Keywords:** Relative Fat Mass, Body Mass Index, Muscle Mass, Obesity Paradox, Cardiometabolic Disease, Heart Failure

## Abstract

**Purpose of Review:**

To position relative fat mass (RFM) as a more accurate, physiologically grounded, and clinically useful alternative to body mass index (BMI) for assessing adiposity and predicting cardiometabolic risk, including heart failure.

**Recent Findings:**

RFM estimates body fat percentage using a sex-specific formula based on waist circumference and height. RFM not only correlates more strongly with fat mass than BMI, but also shows a weaker correlation with muscle mass. This distinction helps reduce lean mass-related confounding in the assessment of adiposity. In clinical studies, RFM has emerged as a robust predictor of incident heart failure, cardiometabolic disease, and all-cause mortality.

**Summary:**

RFM avoids misclassification of adiposity in individuals with high muscle mass and better reflects abdominal adiposity than BMI. As the prevalence of heart failure and other obesity-related diseases continues to rise, RFM offers a practical and intuitive tool for assessment of adiposity and heart failure risk – challenging the long-standing dominance of BMI.

## Introduction

For decades, body mass index (BMI) has been the standard tool for classifying overweight and obesity – not only in clinical practice and guidelines, but also in clinical trial eligibility criteria and public health messaging.

However, BMI does not distinguish between fat mass and lean muscle mass [1, 2]. For example, a sedentary individual and an athletic individual may share the same BMI despite substantial differences in “fat versus muscle” content. Similarly, a bodybuilder or a person with high muscle mass may be misclassified as “overweight” or even “obese.” Additionally, BMI fails to measure the degree of abdominal adiposity and does not account for sex-based differences in fat percentage. These limitations are problematic not only for clinical risk assessment – where actual adiposity, rather than elevated BMI, is more closely linked to the risk of developing heart failure and cardiovascular disease – but also when determining eligibility for clinical trials, potentially excluding or misclassifying individuals based on a flawed metric.

## Relative Fat Mass: A Superior Alternative to BMI

To address these limitations, the relative fat mass (RFM) index was introduced in 2018 by Woolcott and Bergman [3]. RFM is a sex-specific anthropometric measure that estimates body fat percentage using waist circumference and height. The formula, RFM = 64 – (20 × height/waist) + (12 × sex), where sex = 0 for males and 1 for females, was derived and validated in large, diverse population datasets and shows strong correlation with dual-energy X-ray absorptiometry (DEXA) measurements of body fat. In the general population, average RFM values are approximately 25% in males and 35% in females [4], with obesity defined as RFM > 30% in males and > 40% in females [5].

**Fig. 1:**
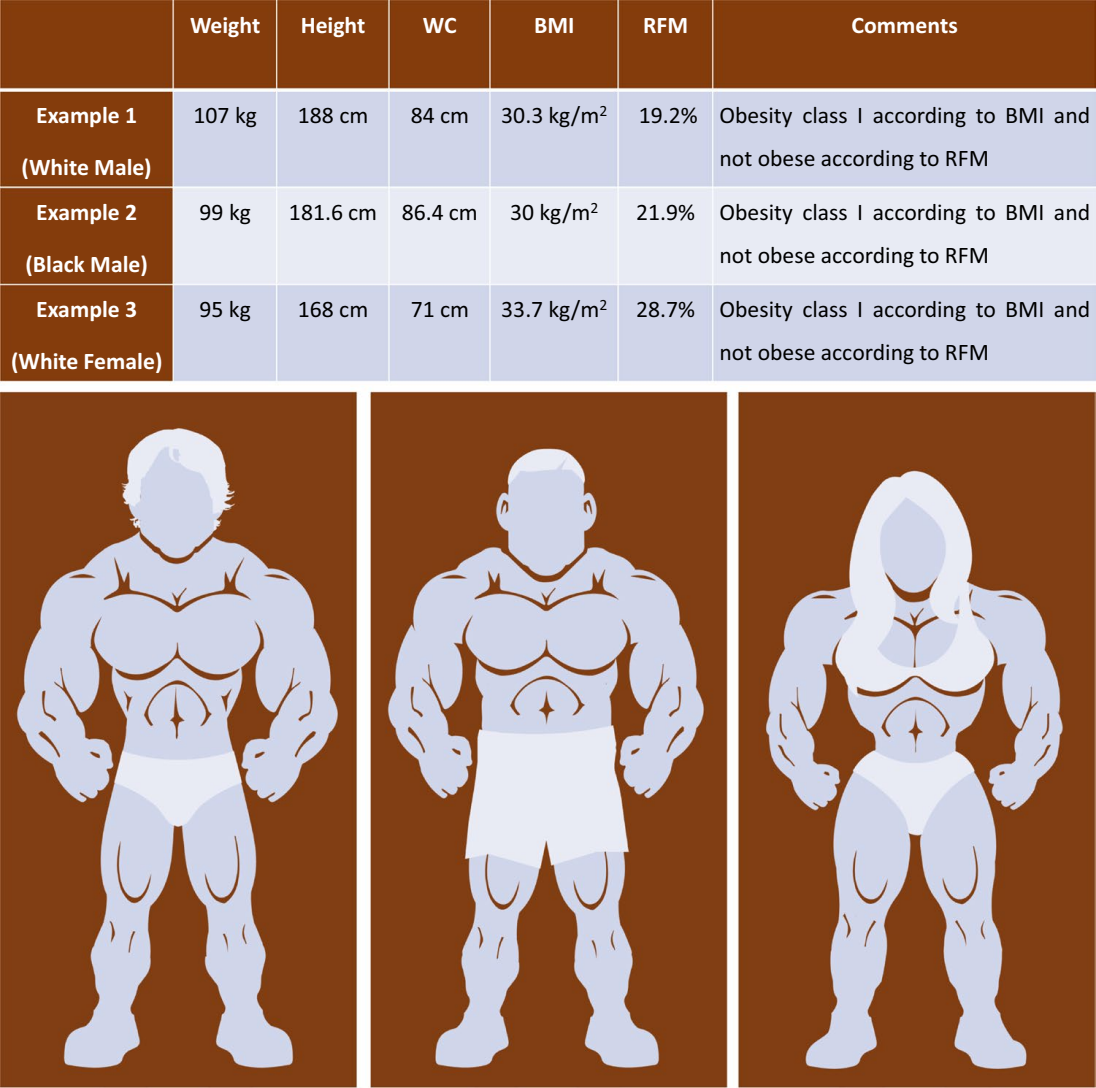
Misclassification of adiposity by body mass index (BMI) in muscular individuals. Three athletic individuals – a White male, a Black male and a White female – are shown with their weight, height, and waist circumference (WC). While BMI (based on weight and height) classifies them as obese, relative fat mass (RFM, based on WC and height) does not. BMI-defined obesity is ≥ 30 kg/m^2^ in both sexes and RFM-defined obesity is > 30% in males and > 40% in females. The images presented are original creations and are used solely for educational and illustrative purposes under fair use. Copyright held by Navin Suthahar, © 2025

Notably, RFM offers two advantages of particular relevance over BMI. First, as RFM is calculated using waist circumference, it provides a more accurate estimate of abdominal fat mass. Second, by excluding body weight from its formula, RFM reduces confounding by muscle mass in the estimation of obesity-related risk [6] – which is an important limitation of BMI (Fig. 1).

## Clinical Relevance of RFM: Risk Prediction and the Obesity Paradox

In Table 1, we summarize key studies highlighting the superiority of RFM over BMI in predicting incident cardiometabolic disease including heart failure (HF) [10]. In addition to being a robust predictor of incident HF and other cardiometabolic outcomes, the use of RFM may also help resolve the “obesity paradox” commonly observed with BMI.

**Table 1:** Key studies highlighting the predictive value of RFM for incident cardiometabolic disease and mortality in the general population

Cohort/Trial	Primary Outcome	Comments
General Population • PREVEND Study • LifeLines Study • Rotterdam Study	Incident type-2 diabetes	Relative fat mass (RFM) was an excellent predictor of incident type-2 diabetes in the general population, and performed better than body mass index (BMI), waist circumference (WC) and waist-to-hip ratio (WHR) [4]
General Population • Lookup 7 + Study	Prevalent hypertension, diabetes and hyperlipidaemia	RFM was an excellent predictor of hypertension and diabetes in elderly individuals, and generally performed better than BMI [7]
General Population • PREVEND Study	Incident heart failure (HF)	RFM was an excellent predictor of incident HF in the general population, and performed better than BMI, WC and WHR [8]
General Population • LifeLines Study	Incident cardiovascular disease (CVD)	RFM was the most consistent predictor of incident CVD and its subtypes in the general population [9]
General Population • PREVEND Study	All-cause mortality	RFM was a stronger predictor of mortality than BMI, and its usage showed no evidence supporting the “obesity-survival paradox” in the general population [6]

This table is reproduced with permission from Suthahar N, et al. *Nature Reviews Endocrinology*; 2025 Jul;21(7):393–394. PREVEND, Prevention of Renal and Vascular End-stage Disease

A likely reason, why this paradox arises, is because BMI is a composite measure reflecting both fat mass and lean muscle mass. Since higher muscle mass is associated with reduced mortality [11, 12] and a higher fat mass with increased mortality [6], BMI — particularly when elevated due to muscle mass — can misleadingly suggest that higher BMI is protective/not deleterious.

Indeed, the BMI-mortality paradox has also been extensively reported in patients with HF (particularly among those with reduced ejection fraction), where a higher BMI has been associated with better survival outcomes. However, this paradox may simply reflect the inability of BMI to distinguish between protective muscle [13] and harmful fat accumulation. Clearly, a patient with HF and preserved muscle mass (higher BMI) would be expected to have better outcomes than one with significant muscle loss (lower BMI) [14]. In such scenarios, usage of RFM, which does not include body weight in its calculation, might help minimize the confounding effect of muscle mass and provide a more accurate assessment of adiposity-related risk.

## Conclusion

In summary, RFM maintains the practical advantages of BMI by requiring two simple non-invasive measurements, while addressing its most significant limitations. By preserving the simplicity and relatability needed for real-world use [10], RFM combines (patho)physiological relevance with clinical practicality. The ability of RFM to reflect abdominal fat distribution, limit the confounding effect of muscle mass, perform consistently across diverse outcomes, and its ease of implementation position it as a strong candidate to replace BMI in routine care.

## Data Availability

No datasets were generated or analysed during the current study.
